# DPP-4 inhibitor sitagliptin prevents inflammation and oxidative stress of heart and kidney in two kidney and one clip (2K1C) rats

**DOI:** 10.1186/s13098-015-0095-3

**Published:** 2015-11-25

**Authors:** Md. Ashraful Alam, Mohammed Riaz Hasan Chowdhury, Preeti Jain, Md. Abu Taher Sagor, Hasan Mahmud Reza

**Affiliations:** Department of Pharmaceutical Sciences, School of Life Sciences, North South University, Bashundhara, Dhaka, Bangladesh

**Keywords:** Sitagliptin, Fibrosis, Inflammation, Oxidative stress, Malondialdehyde

## Abstract

**Background:**

Hyperglycemia and insulin resistance often develop cardiovascular and nephrological dysfunction in diabetic patients. Sitagliptin is used to treat diabetes and showed potential benefit in lowering increased blood glucose level in diabetes. This investigation reports the effect of sitagliptin treatment on oxidative stress in kidney and heart of 2K1C rats.

**Methods:**

Male Long Evans rats underwent unilateral surgical stenosis of the renal artery [2-kidney-1-clip (2K1C) method]. These animals entered a 4-weeks dosing period with sitagliptin. Blood and urine sampling and organ harvesting were finally performed. Blood plasma, heart, kidney tissues and urine were tested for the assessment of inflammation and oxidative stress in kidney and heart of 2K1C rats after 4 weeks of surgery.

**Results:**

2K1C rats showed cardiac hypertrophy, increased left ventricular wet weight compared to sham which was not significantly altered by sitagliptin treatment. Uric acid and creatinin concentrations were also increased in 2K1C rats. Sitagliptin significantly prevented the elevation of uric acid and creatinin concentration in plasma and urine in this rat model. Oxidative stress markers in plasma such as malondialdehyde (MDA), nitric oxide (NO), and advanced protein oxidation product (APOP) concentrations were increased in the 2K1C rats as compared to sham-operated animals. Increased concentrations of these oxidative stress markers were also normalized by sitagliptin treatment. 2K1C rats also showed increased level of uric acid and creatinine both in plasma and urine; which are also reduced to normal level in sitagliptin treated rats. Moreover, 2K1C surgery initiated inflammatory cell infiltration, increased MPO activity and fibrosis in both heart and kidneys which were further ameliorated by sitagliptin treatment.

**Conclusion:**

Our study suggests that sitagliptin treatment in 2K1C rats prevented inflammation and fibrosis of heart and kidney by ameliorating elevated oxidative stress in heart and kidney tissues.

## Background

Congestive heart failure and kidney dysfunction are major causes of mortality and morbidity in patient suffering diabetes worldwide [1, 2]. These secondary complications are frequently seen in most of the diabetes patients and become a public health concern now a day. Myocardial remodeling occurs during the progression of cardiomyopathy in diabetes and possesses an important role in the pathophysiology of hypertensive disease  [3]. Myocardial cell apoptosis, necrosis, hypertrophy and extension have been noted during the progression of myocardial remodeling and cardiac hypertrophy [4, 5]. Moreover, myocardial extracellular matrix and collagen fiber deposition are also found in hypertrophied heart both in experimental animals [6] and in clinical experiments [7]. Free radical mediated oxidative stress is considered as a key player in cardiaomyocyte hypertrophy which can be prevented by antioxidant treatment [8, 9]. The sources of reactive free radicals in heart are mitochondrial electron transport chain, inducible nitric oxide synthase, NADPH oxidase and xanthine oxidase [10]. Oxidative stress mediated insult in the mayocardium also attract inflammatory cells [11]. Several reports also suggest that NADPH oxidase containing neutrophil infiltration in myocardium is mainly responsible for free radicals mediated oxidative stress in heart [9, 12].

Plasma renin release and Ang II activity are increased in case of kidney ischemia [13]. Type 2 diabetes patients often suffer hypertension due to the over activity of renin angiotensin system. Renin angiotensin system plays a crucial role in many pathophysiology of cardiac oxidative stress and myocardial remodeling in diabetes [14]. The two-kidney one-clip (2K1C) renovascular hypertension model animal showed elevated circulating levels of Ang II with high Ang II concentrations in the cortical tissue of the clipped and nonclipped kidneys [15, 16]. Moreover, cross talk between free radicals production and ang-II mediated hypertrophied response in heart is well documented and reviewed recently [8]. Previous studies have reported that angiotensin II (Ang II) stimulates the production of reactive oxygen species (ROS) such as superoxides [17, 18]. In experimental 2K1C hypertension, the overproduction of reactive oxygen species (ROS) may lead to oxidative stress and increased lipid peroxidation [19, 20]. Increased ANG II activity and oxidative stress further triggers the deposition of extracellular matrix and fibrosis in heart and kidney of 2K1C model animal [21, 22].

Dipeptidyl peptidase-4 (DPP-4) inhibitors are used for the treatment of the Type 2 diabetes mellitus (T_2_DM). Sitagliptin belongs to DPP-4 inhibitors group that prevent the degradation of insulinotropic incretin glucagon like peptide (GLP-1), without producing hypoglycemia [23]. DPP-4 inhibitors also preserves islet function in both type 1 and type 2 diabetes animal models and increases pancreatic insulin content, through an increase in proliferation, neogenesis, and apoptosis resistance of beta cells [24]. Previous studies suggest that DPP-4 inhibitors prevent cardiac diastolic dysfunction and ameliorate glomerulopathy in insulin-resistant Zucker obese rats [25, 26]. Improved endothelium-dependent relaxation function in renal arteries, restored renal blood flow and reduced systolic blood pressure was observed in spontaneously hypertensive rats by 2 weeks treatment with sitagliptin [27]. However, sitagliptin appears to limit the blood pressure lowering effect of enalapril in patients with metabolic syndrome [28]. Some other studies also demonstrated the beneficial impact of sitagliptin on diabetic nephropathy [29–31]. Therefore, it is of particular interest to examine whether DPP-4 inhibitor can suppress oxidative stress and inflammation in heart and kidney of two kidney one clip (2K1C) rats.

## Methods

### Chemicals and reagents

Sitagliptin were obtained from Beximco Pharmaceuticals Limited (Bangladesh) as gift sample. Thiobarbituric acid (TBA) was purchased from Sigma Chemical Company (USA). Trichloroacetic acid (TCA) was purchased from J.I. Baker (USA). Alanine aminotransferase (ALT), aspartate aminotransferase (AST), alkaline phosphatase (ALP), and uric acid (UA) assay kits were obtained from DCI diagnostics (Budapest, Hungary). All other chemicals and reagents used were of analytical grade.

### Animal’s surgery and treatment

Twelve to fourteen weeks old, 24 Long Evans male rats (170–230 g) were obtained from Animal production unit of Animal House at Department of Pharmaceutical Sciences, North South University and were kept in ordinary cages at room temperature of 25 ± 3 °C with a 12 h dark/light cycles with food water ad libitum. They have free access to food and water, according to the study protocol approved by Ethical Committee of Department of Pharmaceutical Sciences, North South University for animal care and experimentation. To study the effects of sitagliptin, rats were equally divided into four groups (six rats in each group): Sham, Sham + sitagliptin, 2K1C and 2K1C + sitagliptin. Rats were subjected to unilateral clipping of the renal artery to produce two kidney one clip (2K1C) model rats. In brief, a left kidney was exposed via laparotomy. The left renal artery was separated from the left renal vein and a silk ligature placed around the renal artery. A 23-gauge injection needle was placed into the ligature, parallel to the renal artery, the ligature tied and the needle carefully removed. Visual assessment of kidney perfusion was performed before closing the wound. Sham-operated rats underwent the same procedure, but kidneys were only mobilized and renal vessels were only separated instead of being partially ligated. All procedures were performed under intraperitoneal injection of ketamine anesthesia. Animals were transferred to the housing facility and monitored once they recovered from anesthesia. After surgery, rats were received normal food and water for 28 days. Sham + sitagliptin and 2K1C + sitagliptin groups received sitagliptin (100 mg/kg, daily) by oral gavaging. Animals were checked for the body weight and water intake on a daily basis. After 4 weeks of treatment with or without sitagliptin, the rats were placed in metabolic cages, and urine was collected for 24 h. The urine volume was measured for every rat. After 28 days of the last treatment, all the animals were weighed, sacrificed, collected the blood and organs like heart, kidney, spleen and liver. Immediately after collection of these tissues and organs, they are weighed and stored at −20 °C for further analysis.

### Assessment of AST, ALT and ALP activities

Liver marker enzymes (alanine aminotransferase (ALT), aspartate aminotransferase (AST), and alkaline phosphatase (ALP) were estimated in plasma by using Diatech diagnostic kits for AST, ALT and ALP (Hungary) according to the manufacturer’s protocol. Uric acid and creatinine were also measured using Diatech diagnostic kits for uric acid and creatinin (Hungary) according to the manufacturer’s protocol.

### Assessment of oxidative stress markers

For determination of oxidative stress markers, heart and kidney tissue was homogenized in 10 volumes of Phosphate buffer containing (pH 7.4) and centrifuged at 12,000×*g* for 30 min at 4 °C. The supernatant was collected and used for the determination of protein and enzymatic studies as described below.

### Estimation of lipid peroxidation product malondialdehyde (MDA)

Plasma concentrations of malondialdehyde are an index of lipid peroxidation and oxidative stress. Lipid peroxidation in heart and kidney were estimated colorimetrically measuring malondialdehyde followed by previously described method [32]. In brief, 0.1 mLentrated Acetic Acid-HCl reagent (thiobarbituric acid 0.37 %, 0.25 N HCl and 15 % TCA) and placed in water bath for 15 min and cooled. The absorbance of clear supernatant was measured against reference blank at 532 nm.

### Estimation of nitric oxide (NO)

NO was determined according to the method described by Tracy et al. as nitrate and nitrite [33]. In this study, Griess-Illosvoy reagent was modified by using naphthyl ethylene diamine dihydrochloride (0.1 % w/v) instead of 1-napthylamine (5 %). The reaction mixture (3 mL) containing tissue homogenates (2 mL) and phosphate buffer saline (0.5 mL) was incubated at 25 °C for 150 min. A pink colored chromophore was formed which was measured at 540 nm.

### Estimation of advanced protein oxidation products (APOP)

Determination of APOP levels was performed by modification of the method of Witko-Sarsat [34] and [35] Tiwari. Two mL of plasma was diluted 1:5 in PBS: 0.1 mL of 1.16 M potassium iodide was then added to each tube, followed by 0.2 mL acetic acid after 2 min. The absorbance of the reaction mixture was immediately read at 340 nm against a blank containing 2 mL of PBS, 0.1 mL of potassium iodide (KI), and 0.2 mL of acetic acid. The chloramine-T absorbance at 340 nm being linear within the range of 0–100 mmol/L, APOP concentrations were expressed as μmol L^−1^ chloramine-T equivalents.

### Estimation of catalase (CAT) activity

CAT activities were determined by the method of Chance and Maehly [36, 37] with some modifications. The reaction solution of CAT activities contained: 2.5 ml of 50 mmol phosphate buffer (pH 5.0), 0.4 mL of 5.9 mmol H_2_O_2_ and 0.1 mL enzyme extract. Changes in absorbance of the reaction solution at 240 nm were determined after 1 min. One unit of CAT activity was defined as an absorbance change of 0.01 as units/min.

### Estimation of myloperoxidase (MPO) activity

MPO activity was determined by a dianisidine-H_2_O_2_ method [38], modified for 96-well plates. Briefly, plasma samples (10 μg protein) were added in triplicate to 0.53 mM *o*-dianisidine dihydrochloride (Sigma) and 0.15 mM H_2_O_2_ in 50 mM potassium phosphate buffer (pH 6.0). The change in absorbance was measured at 460 nm. Results were expressed as units of MPO/mg protein.

### Histopathalogical determination

For microscopic evaluation heart and kidney tissues were fixed in neutral buffered formalin and embedded in paraffin, sectioned at 5 μm and subsequently stained with hematoxylin and eosin (H & E) to evaluate inflammatory cell infiltration. Sirius red staining was also performed to evaluate the fibrosis in heart and kidney. Moreover, Prussian blue staining was done to determine the iron deposition in tissues. Sections were studied under light microscope at 40× magnifications.

### Statistical analysis

The values are expressed as mean ± standard deviation (SD). The results were evaluated by using the Two-way ANOVA followed by Bonferroni test using Graph Pad Prism Software, USA, version 6. Statistical significance was considered as *p* < 0.05 in all cases.

## Results

### Effect of sitagliptin on body weight, food and water intake in 2K1C rats

Body weight of each rat was recorded every day during the experiment, and % change was calculated for all groups. It was found that the body weight increased consistently in sitagliptin treated rats group, which is denoted that the treatment have no effect on the body weight. On the other hand sham + sitagliptin group showed no variation in their body weight. Water intake was not changed significantly among the groups.

### Effect of sitagliptin on organ wet weight in 2K1C rats

Table 1 shows the effect of various treatments on the rats’ organs wet weight. The wet weight of heart and kidney was increased in the 2K1C rats when compared with sham rats. Left ventricular wet weight was increased significantly compared to sham rats. However, sitagliptin treatment showed no significant change in the wet weight of the left ventricle of heart in the 2K1C rats. Neither 2K1C challenge and nor sitagliptin treatment in rats have changed the right ventricular wet weight significantly compared to sham rats. 2K1C rats also showed slight decrease in liver wet weight, however, sitagliptin treatment did not altered the wet weight of the liver compared to 2K1C rats. Another crucial finding in this study was that 2K1C rats showed increased kidney wet weight compared to sham rats which were unaltered by sitagliptin treatment (Table 1). Furthermore, 2K1C rats showed increased spleen wet weight compared to sham rats which were reduced by sitagliptin treatment in 2K1C rats.

**Table 1:** Effect of sitagliptin on body weight, food and water intake and organ weight of 2K1C rats

Parameter	Sham	Sham + Sitagliptin	2K1C	2K1C + Sitagliptin	*p* values		
					2K1C	Treatment	Interaction
Initial body weight (gm)	178.07 ± 6.80	218.40 ± 7.49	214.26 ± 3.39	231.17 ± 11.42	0.0023	0.0073	0.1671
Final body weight (gm)	224.75 ± 14.86	229.55 ± 4.77	269.10 ± 10.56	251.47 ± 12.66	0.5833	0.0095	0.3415
Water intake/d	18.61 ± 1.10	15.69 ± 0.43	20.64 ± 0.72	22.58 ± 0.80	0.5529	<0.0001	0.0074
Liver wet weight	3.24 ± 0.10	3.59 ± 0.24	3.22 ± 0.24	3.72 ± 0.12	0.0310	0.7663	0.6855
Kidney wet weight	1.40 ± 0.07	1.62 ± 0.06	2.36 ± 0.11	2.50 ± 0.18	0.1382	<0.0001	0.7347
Heart wet weight	1.29 ± 0.07	1.45 ± 0.04	1.59 ± 0.05	1.40 ± 0.08	0.0576	0.8114	0.0106
Left ventricle wt.	1.03 ± 0.03	1.06 ± 0.02	1.24 ± 0.07	1.16 ± 0.07	0.0081	0.6402	0.3089
Right ventricle wt.	0.16 ± 0.02	0.23 ± 0.01	0.24 ± 0.02	0.20 ± 0.02	0.1808	0.4152	0.0063
Spleen wet weight	1.37 ± 0.16	1.58 ± 0.16	2.39 ± 0.66	2.00 ± 0.17	0.2505	0.0774	0.1647

Values are presented as mean ± SEM. Two way or One way ANOVA with Bonferoni tests were done as post hoc test

Values are considered significance at p < 0.05. (Organ wet weights are expressed as gm/100 gm of body weight)

### Effect of sitagliptin on biochemical parameters AST, ALT and ALP activity

Biochemical measurement of 2K1C rats showed a significant increase in plasma AST, ALT, and ALP activity compared to sham rats (Table 2). Treatment with sitagliptin in 2K1C rats significantly counteracted these increased enzymes activity. In addition, treatment of animals with sitagliptin alone for 4 weeks did not show any significant changes in AST, ALT and ALP enzyme activities compared to the sham rats (Table 2).

**Table 2:** Effect of sitagiliptin on biochemical parameters in plasma and urine of 2K1C rats

Parameters	Sham	Sham + Sitagliptin	2K1C	2K1C + Sitagliptin	*p* values		
					2K1C	Treatment	Interaction
Plasma							
AST (U/L)	26.97 ± 1.25	24.42 ± 0.67	35.27 ± 1.29	27.08 ± 0.58	<0.0001	<0.0001	0.0107
ALT (U/L)	18.32 ± 0.94	20.77 ± 0.76	35.27 ± 1.29	24.35 ± 0.42	0.0002	<0.0001	<0.0001
ALP (U/L)	43.30 ± 1.22	40.83 ± 1.92	56.23 ± 2.49	50.97 ± 1.72	0.0546	<0.0001	0.4697
MDA (nmol/mL)	5.03 ± 0.20	6.58 ± 0.32	8.13 ± 0.24	3.72 ± 0.12	0.0004	<0.0001	0.0630
NO (nmol/mL)	2.82 ± 0.12	3.30 ± 0.31	7.78 ± 0.28	5.20 ± 0.21	0.0003	<0.0001	<0.0001
APOP (ng/mL)	102.45 ± 4.30	129.53 ± 4.73	304.96 ± 10.55	223.33 ± 8.97	0.0019	<0.0001	<0.0001
CATALASE (U/L)	5.42 ± 0.08	5.37 ± 0.13	3.43 ± 0.20	4.05 ± 0.29	0.1532	<0.0001	0.0963
Uric acid (mg/dL)	4.93 ± 0.54	6.24 ± 0.72	12.18 ± 1.15	8.41 ± 0.75	<0.0001	0.1535	0.0070
Creatinin (mg/dL)	1.36 ± 0.10	1.56 ± 0.05	2.30 ± 0.20	1.46 ± 0.31	0.0158	0.1163	0.0446
Urine							
Uric acid (mg/dL)	6.25 ± 0.18	4.18 ± 0.25	8.25 ± 0.33	6.78 ± 0.23	<0.0001	<0.0001	0.2536
Creatinin (mg/dL)	10.76 ± 0.54	5.60 ± 0.82	18.90 ± 2.73	7.03 ± 1.18	0.0062	<0.0001	0.0446

Values are presented as mean ± SEM. Two way or One way ANOVA with Bonferoni tests were done as post hoc test

*APOP* advanced protein oxidation product, expressed as nmol/mL equivalent to Chloramine-T

Values are considered significance at p < 0.05

### Effect of sitagliptin on oxidative stress markers and antioxidant enzymes

To determine the oxidative stress in our study, we evaluated the malondialdehyde (MDA), nitric oxide and advanced protein oxidation product (APOP) content in plasma, heart and kidneys. 2K1C rats showed a higher concentration of lipid peroxidation product MDA in plasma, heart and kidney (Tables 2, 3). Additionally, sitagliptin treatment in 2K1C rats significantly reduced the level of lipid peroxides compared to 2K1C group in plasma, heart and kidney. 2K1C rats also showed profound effect on APOP development in plasma and kidney tissues (Tables 2, 3). 2K1C rats showed significantly increase concentration of APOP in plasma and kidney which was normalized due to sitagliptin treatment in 2K1C rats. APOP concentration was unchanged in all groups tested in this study. Nitric oxide measured as nitrate was also increased in plasma, heart and kidney compared to sham rats which were further normalized by sitagliptin treatment in 2K1C group (Tables 2, 3). 2K1C group rats showed decrease in antioxidant enzyme catalase activity compared to the sham group rats (Tables 2, 3). Treatment with sitagliptin to 2K1C significantly counteracted the oxidative stress by restoring the catalase activity to near normal compared to 2K1C group (Tables 2, 3).

**Table 3:** Effect of sitagiliptin on oxidative stress parameters in heart and kidney of 2K1C rats

Parameter	Sham	Sham + Sitagliptin	2K1C	2K1C + Sitagliptin	*p* values		
					2K1C	Treatment	Interaction
Heart							
MDA (nmol/mL)	47.62 ± 1.76	50.38 ± 3.41	77.08 ± 4.88	61.46 ± 4.06	<0.0001	0.1912	0.0087
NO (nmol/mL)	13.08 ± 2.52	9.66 ± 0.42	25.21 ± 2.81	21.86 ± 3.67	0.0002	0.2145	0.9896
APOP (ng/mL)	227.30 ± 32.31	230.48 ± 12.34	237.62 ± 22.28	232.86 ± 36.91	0.8246	0.9780	0.8897
CATALASE (U/L)	7.64 ± 0.31	7.40 ± 0.73	5.48 ± 0.54	7.08 ± 0.60	0.0482	0.2613	0.1338
MPO Activity (U/mg protein)	2.20 ± 0.09	1.90 ± 0.18	3.77 ± 0.17	2.30 ± 0.17	<0.0001	<0.0001	0.0013
Kidney							
MDA (nmol/mL)	101.38 ± 7.84	124.92 ± 6.92	183.00 ± 10.77	133.51 ± 9.85	0.0001	0.1820	0.0010
NO (nmol/mL)	13.89 ± 1.28	11.21 ± 1.81	62.36 ± 5.49	52.59 ± 1.93	<0.0001	0.0594	0.2684
APOP (ng/mL)	391.90 ± 40.11	421.75 ± 24.02	978.57 ± 100.73	348.73 ± 35.44	<0.0001	<0.0001	<0.0001
CATALASE (U/L)	12.72 ± 1.20	10.50 ± 1.50	6.70 ± 0.38	11.08 ± 1.04	0.0234	0.3416	0.0075
MPO Activity (U/mg protein)	1.94 ± 0.28	1.79 ± 0.18	2.80 ± 0.22	1.85 ± 0.09	0.0359	0.0141	0.0646

Values are presented as mean ± SEM. Two way or One way ANOVA with Bonferoni tests were done as post hoc test

*APOP* advanced protein oxidation product, expressed as nmol/mL equivalent to Chloramine-T

Values are considered significance at p < 0.05

### Effect of sitagliptin on uric acid and creatinine concentration in plasma and urine

Uric acid concentration in plasma and urine was increased in 2K1C rats significantly compared to the sham rats. Sitagliptin treatment in these rats lowered the uric acid concentration significantly compared to 2K1C rats group. Sitagliptin treatment in sham rats did not change uric acid concentration in plasma compared to sham rats.

2K1C rats also showed increased creatinin concentration both in plasma and urine significantly compared to sham rats. Sitagliptin treatment normalized the creatinin concentration in plasma and urine of 2K1C rats.

### Effect of sitagliptin on inflammation and fibrosis markers in heart and kidneys

**Fig. 1:**
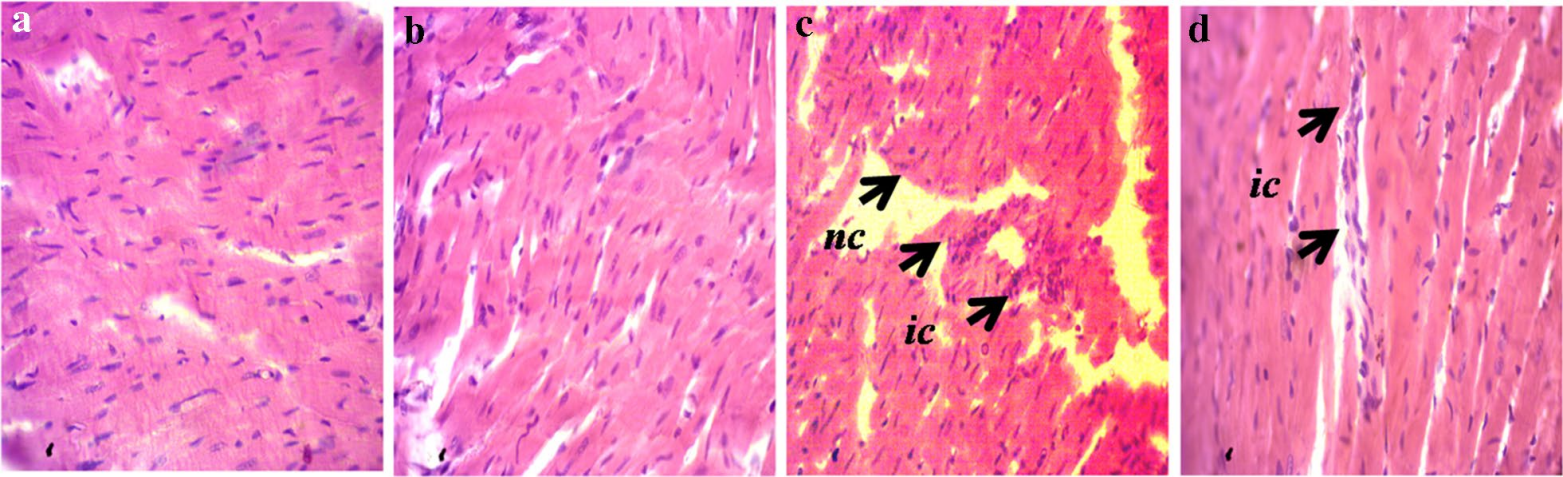
Effect ofsitagliptin on cardiac inflammation in 2K1C model rats. **a** Sham; **b** Sham + Sitagliptin; **c** 2K1C and **d** 2K1C + Sitagliptin. No inflammatory process was observed in Sham animals (**a**) and Sham + Sitagliptin (**b**). Observe the increase of inflammatory cells in the *left ventricle* of heart of 2K1C rats (**c**, *arrows*). No/less inflammatory process can be observed in *left ventricle* of heart of all 2K1C animals treated with sitagliptin (**d**). Magnification ×40. H & E staining. *ic* inflammatory cells, *nc* necrotic site

**Fig. 2:**
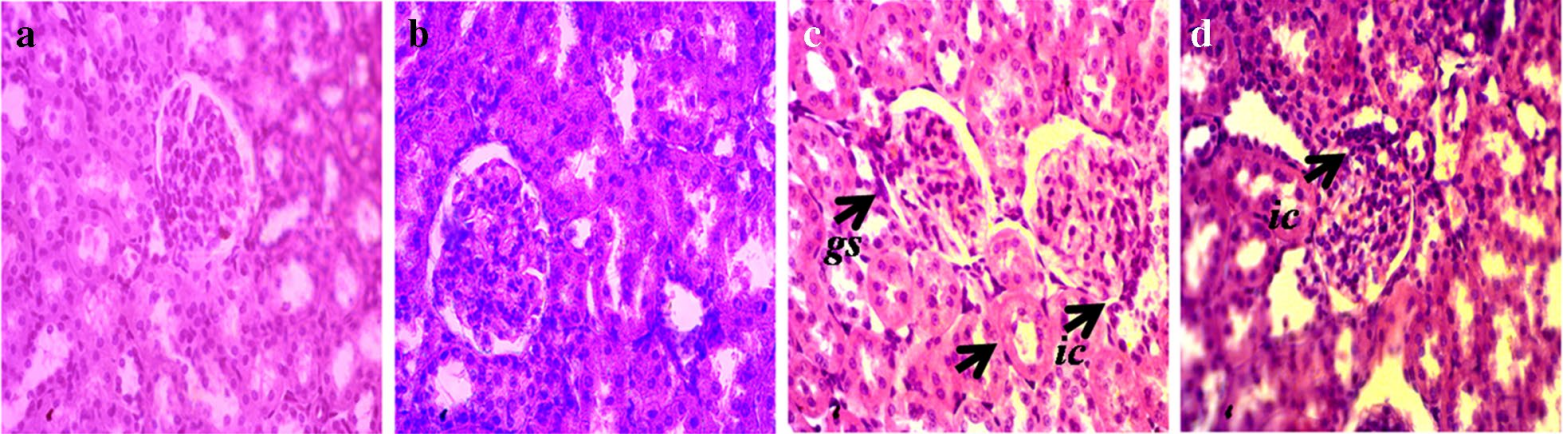
Effect of sitagliptin on kidney inflammation in 2K1C model rats. **a** Sham; **b** Sham + Sitagliptin; **c** 2K1C and **d** 2K1C + Sitagliptin. No inflammatory process was observed in Sham animals (**a**) and Sham + Sitagliptin (**b**). Observe the increase of inflammatory cells in the kidneys of 2K1C (**c** *arrows*). Glomerular sclerosis, tubular atrophy with accompanying moderate interstitial fibrosis and infiltration by mononuclear cells was also found. No/less inflammatory process can be observed in right kidney of 2K1C treated with sitagliptin (**d**). Magnification ×40. H & E staining. *ic* inflammatory cells, *gc* glomerular sclerosis

**Fig. 3:**
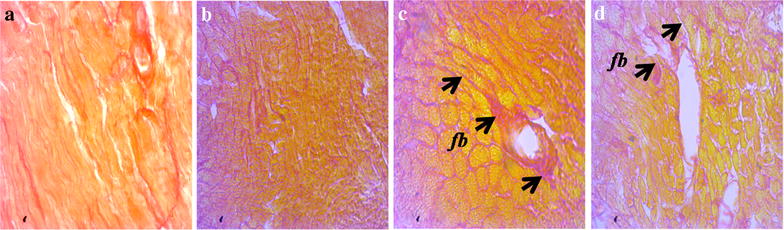
Effect of sitagliptin on cardiac fibrosis in 2K1C model rats. Picrosirius red staining for fibrosis. **a** Sham; **b** Sham + Sitagliptin; **c** 2K1C and **d** 2K1C + Sitagliptin. No pathological collagen tissue deposition was observed in Sham animals (**a**) and Sham + Sitagliptin (**b**). Observe the greater collagen deposition in the *left ventricle* of heart of 2K1C rats (**c** *arrows*). No/less collagen tissue deposition can be observed in *left ventricle* of heart of all 2K1C animals treated with sitagliptin (**d**). Magnification ×40. *fb* fibrosis

**Fig. 4:**
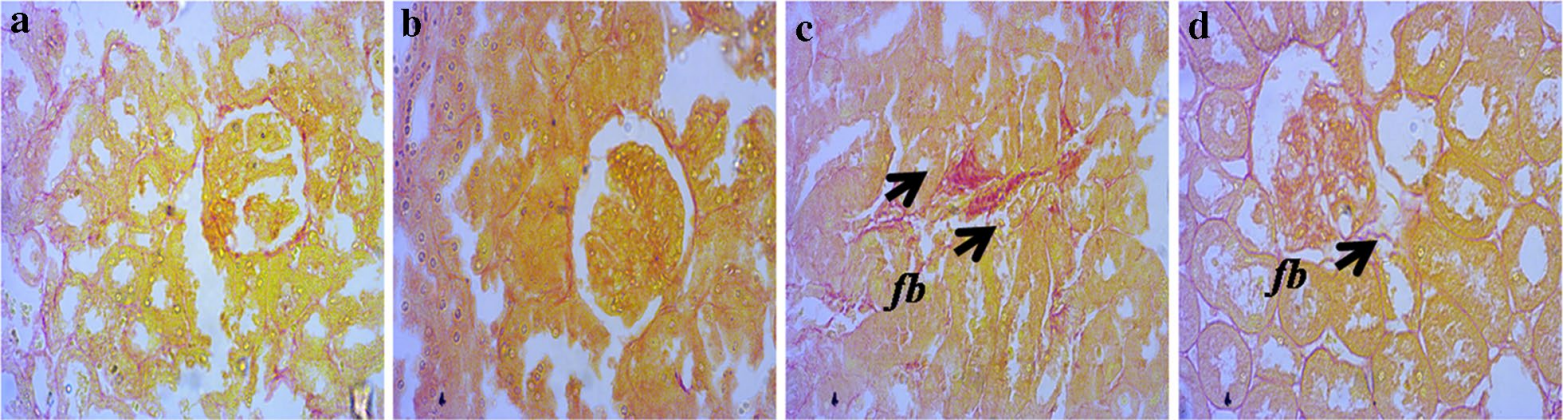
Effect of sitagliptin on kidney fibrosis in 2K1C model rats. **a** Sham; **b** Sham + Sitagliptin; **c** 2K1C and **d** 2K1C + Sitagliptin. No pathological collagen tissue deposition was observed in Sham animals (**a**) and Sham + Sitagliptin (**b**). Observe the greater collagen deposition in the *left* kidney of 2K1C rats (**c** *arrows*). No/less collagen tissue deposition can be observed in right kidney of all 2K1C animals treated with sitagliptin (**d**). Magnification ×40. *fb* fibrosis

**Fig. 5:**
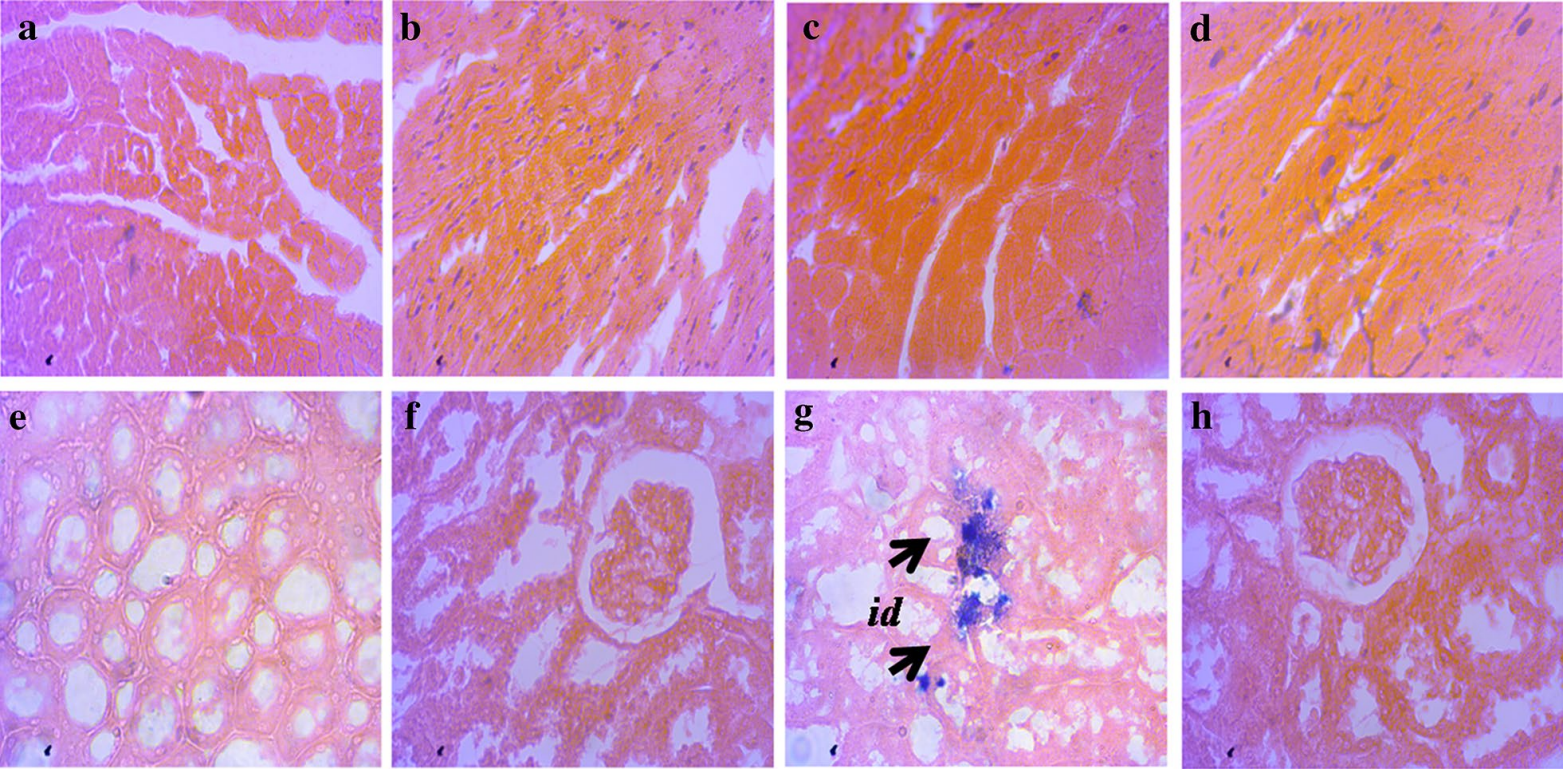
Effect of sitagliptin on cardiac and kidney iron deposition in 2K1C model rats. **a**, **e** Sham; **b**, **f** Sham + Sitagliptin; **c**, **g** 2K1C and **d**, **h** 2K1C + Sitagliptin. No pathological iron deposition was observed in *left ventricle* of heart (*upper panel*). Moreover, no pathological iron deposition was observed in kidneys of Sham animals (**e**) and Sham + Sitagliptin (**f**). Observe the greater iron deposition in the kidney of 2K1C rats (**g** *arrows*). No/less iron deposition can be observed in kidney of all 2K1C animals treated with sitagliptin (**h**). Magnification ×40. *id* iron deposition

Inflammation was seen in rats of 2K1C group compared to sham rats. To determine inflammation in tissues, we measured myloperoxidase (MPO) activity in heart and kidney tissues. 2K1C group rats showed increased MPO activity both in heart and kidney compared to sham rats. Sitagliptin treatment significantly normalized the MPO activity in 2K1C rats compared to sham rats. These data are further supported by the histological assessment of tissue sections of heart and kidneys. Necrotized tissue scar and ballooning of the cardiomyocytes were also seen in heart of 2K1C rats (Fig. 1). Sitagliptin treatment significantly attenuated the inflammatory cell infiltration and necrosis in the heart tissues of 2K1C rats (Fig. 1). Moreover, sham rats and sham rats treated with sitagliptin showed no inflammatory cells infiltration in left ventricle of heart. Massive serge of inflammatory cells was found in the glomerular part of 2K1C rats kidney sections stained with H & E. Glomerulosclerosis was evident, and the interstitium showed patchy infiltrates of mononuclear cells as well as fibrosis (Fig. 2). However, no significant difference could be detected between sham rats and 2K1C rats treated with sitagliptin, demonstrating complete healing of the nephritic changes (Fig. 2). Cardiac and kidney fibrosis were evaluated histologically by visualizing the red color collagen fibers deposition using Sirius red staining process. Sham rats and sham rats treated with sitagliptin showed no/less deposition of collagen fibers in heart and kidneys. However, 2K1C rats showed massive collagen deposition both in heart and kidney which was further attenuated by sitagliptin treatment (Figs. 3, 4). Furthermore, free iron deposition was also seen in kidney sections of 2K1C rats which were ameliorated by sitagliptin treatment (Fig. 5). However, no iron deposition occurred in left ventricular section of all groups tested in this study.

## Discussion

This study demonstrated that sitagliptin treatment prevented the oxidative stress, inflammatory cell infiltration and fibrosis in heart and kidney of 2 kidney one clip (2K1C) rats. Previous studies suggest that, two-kidney, one-clip (2K1C) rat model experience a decreased renal perfusion pressure which causes the kidney to overproduce renin and leads to a continual activation of the renin–angiotensin–aldosterone axis [39]. Renal artery constriction, usually from atherosclerotic or fibromuscular dysplastic renal disease may develop such condition in human [40].

Oxidative stress due to excess generation of ROS plays an important role in producing tissue damage to the organ or increased the inflammatory response which ultimately stimulates the production of pro-fibrogenic mediators and initiate fibrogenesis. Lipid peroxidation, arising from the reaction of free radicals with lipids, has been linked with altered membrane structure and enzyme inactivation. Its end products are measured as TBARS, lipid hydroperoxides and conjugated dienes [41]. In our study, all 2K1C groups showed increased serum malon dialadehyde (MDA) indicating increased production of toxic aldehydes in rats as previously reported [41]. This enhancement of lipid peroxidation products might be due to increased tissue damage, free radical production and decreased hydrolysis of lipid peroxides [41]. APOP level was also increased in our study in 2K1C rats. We explored the protective mechanisms of sitagliptin by studying markers of oxidative stress and inflammation. Furthermore, sitagliptin treatment alone significantly enhanced the antioxidant enzyme activities and inhibited lipid peroxidation as compared to the sham rats. These findings support the premise that sitagliptin can guard against the sequences of oxidative stress.

Nitric oxide (NO) is sometime considered as another mediator of the oxidative stress. Nitric oxide may convert into peroxinitrile which is much more dangerous than superoxide itself and causes more cellular damage in presence of superoxide free radicals. However, NO plays a significant role in the regulation of blood pressure and that impaired NO bioactivity is an important component of hypertension [42]. In our study, we also found that NO level increased in plasma of 2K1C rats which was normalized by sitagliptin treatment. Previous study suggest that endothelial nitric oxide synthase (eNOS) and inducible nitric oxide expression were increased in 2K1C rats [43]. Previous studies have also shown that iNOS isoforms are able to generate superoxide anions independent of NO production [44]. In our study, we also observed that AST, ALT, and ALP activities were increased significantly in 2K1C rats. Sitagliptin treatment normalized these parameters which signify the overall improvement in health condition of the studied animals.

In cardiovascular remodeling, reactive free radical species mediated oxidative stress and infiltration of inflammatory cells have been noticed in remodeled heart and implicates myocardial hypertrophy, fibrosis, conduction abnormalities and endothelial dysfunction which ultimately leading to heart failure [45, 46]. Moreover, Ang-II promotes cardiac and kidney cell apoptosis and triggers fibrosis by activating the fibroblast cells and other growth mediators like TGF-β [47, 48]. Our study showed that massive collagen was deposited in both heart and kidney tissues. Sitagliptin supplementation attenuated this collagen deposition as well as decreased the inflammation in these tissues.

Development and progression of nephropathy is another complication of diabetes which is primarily evaluated by glomerular hyperfiltration [49]. Moreover, activation of several metabolic pathways such as activation of protein kinase C [50], nonenzymatic glycosylation [51] acceleration of the polyol pathway [52], hexosamine biosynthetic pathway [53], and oxidative stress [54] are also involves in the development of diabetic nephropathy. Further evidences are also pointing to a vital role of the inflammatory process in the development and progression of diabetic nephropathy [55–57]. Diverse inflammatory cells, including macrophages, monocytes, and leukocytes, as well as other molecules, such as chemokines, adhesion molecules, and inflammatory cytokines, namely, tumor necrosis factor alpha (TNF-α) and interleukin-1β(IL-1β) [57–59] are few mediators of the inflammatory responses. If inflammation persists on, certain vascular lesions are aggravated, such as endothelial dysfunction, tissue damage, renal fibrosis, and apoptotic cell death [58, 59]. In our study, the wet weight of remnant kidney is significantly changed in treatment group when compared with sham rats. Moreover, our study suggests that sitagliptin supplementation also improved the uric acid and creatinin level in plasma of 2K1C rats. Previous report suggests that 2K1C rat model showed significant increase in rennin activity and Ang-II in circulating blood and tissues [60, 61]. Ang-II promotes cardiac and kidney growth and in pathological condition this growth may turn into hypertrophy [62]. Moreover, in this model one kidney was clipped and other one set free to serve the whole body. To facilitate the whole body blood purification, this second kidney adjusted itself and grows almost double. Previous report suggests that sitagliptin improves renal dysfunction and reduced glomerular and tubulointerstitial injury and exerts anti-oxidative, anti-apoptotic, and anti-inflammatory effects [63].

Our study revealed the anti fibrotic activity of sitagliptin in heart and kidneys of 2K1C rats. The beneficial effect of sitagliptin was mainly due to the improvement of oxidative stress and inflammation in this rat model. Further research is required to establish clinical benefit of sitagliptin in inflammation and fibrosis in diabetic hypertensive patients.
